# Our Journal Volume Two

**Published:** 1885-03

**Authors:** 


### Editorial.

### OUR JOURNAL, VOLUME TWO.

With the present number of the ATLANTA MEDICAL AND SURGICAL JOURNAL begins the second year of its history under its present management. The occasion offers a favorable opportunity to exchange a few words of congratulation with our friends and patrons.

The first series of the JOURNAL had declined in circulation, in the richness and interest of its contents, and in public approbation until its final cessation seemed inevitable. The prestige of its failures, in addition to the usual causes of doubt of success, rested upon the new enterprise; but, believing that Atlanta should be, and would become, for a large extent of surrounding territory, the centre of medical instruction, of professional activity and scientific progress, as it is already the capital of the manufacturing, commercial, transportational and political activity for so large a section of our country, the proprietors determined to incur the hazard and offer to the medical profession a journal deserving its patronage.

That we have reached the measure of our ambition and hopes we do not pretend, but we have received from kind notices in contemporary journals, from large private correspondence and from other sources, most gratifying assurances that we have attained a large measure of success. The circulation of the JOURNAL has, within the year, more than quadrupled that of its predecessor, and now reaches a constituency scattered over the entire Southern States. We have great pleasure in stating that the financial condition of the JOURNAL is so satisfactory as to absolutely guarantee its permanent publication and progressive improvement.

The editors desire to say a few words to our patrons, many of whom are their personal friends and former pupils. It is our purpose to make the ATLANTA MEDICAL JOURNAL fairly representative of the professional thought and opinions of Southern physicians—to make it the microcosm of southern medical literature. This cannot be done by writing long editorials or didactic essays, but by collecting, *editing* and collating the work of large numbers of intelligent physicians who have opportunities of observing disease, climatic influence, local or general morbific causes, therapeutic facts or opinions from every possible point of observation. To aid us in this purpose, we earnestly solicit from our friends and patrons communications upon any and all subjects of professional interest. We do not ask exhaustive treatises, but prefer brief expressions of opinion, with noted observations, suggestive inquiries, conclusions based upon personal experience, and the frank expression of the thought of the writer on whatever point he may regard as important or interesting. We know many physicians of large experience, of sound judgment and approved skill, who contribute nothing to the literature of the profession, because they think publishers desire elaborate essays. Short articles of a few lines or one or two pages are most welcome. The coöperation of our friends, we hope, will enable us to make the JOURNAL a medium of inter-communication between them, and in a few years the recognized organ of professional opinion throughout the South.

Published in the interest of no school of medicine, no clique or cabal of physicians, for fostering the reputation of no particular man or association, but solely to promote the honor and welfare of the whole guild, we invite the assistance of all who will write in the spirit of our motto: "*Pax et scientia, sed veritas sine timore.*"

The pictorial feature of the JOURNAL has met with such universal favor that we are constrained to continue it. Future numbers will be illustrated by the likenesses of leading members of the profession, living and dead, in this and surrounding States.

